# Treatment of Opioid Use Disorder in Pregnant Women via Telemedicine: A Nonrandomized Controlled Trial

**DOI:** 10.1001/jamanetworkopen.2019.20177

**Published:** 2020-01-31

**Authors:** Constance Guille, Annie N. Simpson, Edie Douglas, Lisa Boyars, Kathryn Cristaldi, James McElligott, Donna Johnson, Kathleen Brady

**Affiliations:** 1 Department of Psychiatry and Behavioral Sciences, Medical University of South Carolina, Charleston; 2 Obstetrics and Gynecology, Medical University of South Carolina, Charleston; 3 Department of Healthcare Leadership and Management, Medical University of South Carolina, Charleston; 4 Department of Pediatrics, Medical University of South Carolina, Charleston

## Abstract

**Importance:**

There are high rates of maternal and newborn morbidity and mortality associated with opioid use disorder (OUD). Integrating OUD treatment in obstetric practices for pregnant and postpartum women via telemedicine can increase access to care and reduce the consequences of OUD. Evaluation of this care delivery model, however, is needed before widespread adoption.

**Objective:**

To compare maternal and newborn outcomes among pregnant women with OUD receiving care via telemedicine vs in person.

**Design, Setting, and Participants:**

A nonrandomized controlled trial including 98 women receiving perinatal OUD treatment in 4 outpatient obstetric practices by telemedicine or in person and followed up until 6 to 8 weeks post partum was conducted from September 4, 2017, to December 31, 2018. Logistic regression with propensity score adjustment was applied to reduce group selection bias and control for potentially confounding variables.

**Interventions:**

Participants were seen weekly for 4 weeks, every 2 weeks for 4 weeks, and monthly thereafter and provided relapse prevention therapy and buprenorphine.

**Main Outcomes and Measures:**

The outcomes were retention in treatment, defined as uninterrupted addiction treatment during pregnancy through 6 to 8 weeks post partum; urine drug screen results at delivery and 6 to 8 weeks post partum; and a neonatal abstinence syndrome (NAS) diagnosis collected via electronic health records.

**Results:**

The mean (SD) age of the 98 pregnant women was 30.23 (5.12) years. Of these, 41 of 44 women (93.2%) in the telemedicine group and 48 of 54 women (88.9%) in the in-person group chose to continue treatment in the program after an initial evaluation. After propensity score weighting and doubly robust estimation, no significant differences were found between groups in retention in treatment at 6 to 8 weeks post partum (telemedicine: 80.4% vs in person: 92.7%; treatment effect, −12.2%; 95% CI, −32.3% to −4.4%). Similarly, after propensity score weighting and doubly robust estimation, there were no significant group differences in rates of NAS (telemedicine: 45.4% vs in person: 63.2%; treatment effect, −17.8%; 95% CI, −41.0% to 8.9%).

**Conclusions and Relevance:**

In this nonrandomized controlled trial, virtually integrated OUD care in obstetric practices produced similar maternal and newborn outcomes compared with in-person care. These findings may have important public health implications for combatting the opioid crisis and its consequences on pregnant women and their families. Future large randomized clinical trials are needed.

**Clinical Trial Registration:**

ClinicalTrials.gov identifier: NCT04049032

## Key Points

**Question:**

Is opioid use disorder treatment received via telemedicine in obstetric practices associated with similar maternal and newborn outcomes compared with opioid use disorder treatment received in person in obstetric practices?

**Findings:**

In this nonrandomized controlled trial including 98 pregnant women with opioid use disorder, there were no statistically significant differences in rates of retention in treatment between women receiving opioid use disorder treatment via telemedicine vs in person (80.4% vs 92.7%). These findings were also apparent in newborns with neonatal abstinence syndrome (telemedicine: 45.4% vs in person: 63.2%).

**Meaning:**

Telemedicine may provide a scalable solution to making lifesaving treatment available to pregnant women to reduce the maternal morbidity and mortality associated with opioid use disorder and improve maternal and child health.

## Teaser

This nonrandomized controlled trial evaluates treatment of opioid use disorder in pregnant women via telemedicine vs in-person care.

## Introduction

From 1999 to 2014, the number of pregnant women with opioid use disorder (OUD) in the United States more than quadrupled, increasing from 1.5 to 6.5 cases per 1000 hospital births.^1^ The increasing prevalence of perinatal OUD and its consequences for pregnant women and infants are of increasing public health concern owing to the significant morbidity and mortality associated with this chronic disease.^2^ One of the well-known consequences of opioid use in pregnancy is neonatal abstinence syndrome (NAS), which is the signs and symptoms of withdrawal that infants develop after in utero exposure to substances, including opioids. From 2004 to 2014, the rate of NAS increased from 1.5 to 8.0 per 1000 hospital births.^3^ The increasing rates of NAS have disproportionately occurred in rural and impoverished communities and are associated with counties that have high rates of long-term unemployment and a lack of mental health clinicians.^4^

Although integrated obstetric and addiction care is associated with improvements in maternal and newborn health,^5,6^ it can be difficult to achieve, particularly in rural areas, and nationally there remains a dearth of treatment programs for pregnant women with substance use disorders.^7^ Pharmacotherapy, such as buprenorphine or methadone, is considered part of the standard of care for pregnant women with OUD.^8^ However, less than one-quarter of pregnant women with OUD will receive substance use treatment, and among those who do, few will receive pharmacotherapy for OUD.^9^ There is a myriad of barriers to care for this population,^10^ but a major factor in the low use of pharmacotherapy is lack of access, particularly in rural areas of the country.^11,12,13^

Telemedicine is a tool to expand the reach of addiction specialists and integrate addiction treatment into obstetric care. However, legislation originally intended to prevent the prescribing of controlled substances via the internet has hindered progress in the use of telemedicine for the treatment of OUD nationally due to an in-person visit requirement.^14^ Current legislation has called for the revising of laws governing the prescribing of controlled substances via telemedicine.^14^ Yet, there are few empirical data to guide these revisions^15,16^ and, to our knowledge, there have been no studies describing a telemedicine program for the treatment of OUD in pregnant women.

The purpose of this study was to use propensity score weighting to compare retention in treatment, substance use, NAS, and length of newborn hospital stay among women who received OUD treatment via telemedicine or in person in obstetric practices. We hypothesized that maternal and newborn outcomes would not differ between those receiving integrated addiction care in person vs through telemedicine.

## Methods

### Participants and Study Design

Participants in this nonrandomized controlled trial were recruited from the prospectively collected data in the Women’s Reproductive Behavioral Health Program at the Medical University of South Carolina. This database contains information about pregnant women who sought treatment for OUD either in person or via telemedicine at their obstetrician’s office. Collection of routine clinical assessments are completed at each visit for the purpose of clinical care and deidentified for data analysis. This study was approved by the Medical University of South Carolina’s Institutional Review Board, and a waiver of written and oral informed consent was granted because data were from a database and deidentified. This study followed the Transparent Reporting of Evaluations With Nonrandomized Designs (TREND) reporting guideline.

Consecutive cases of pregnant women with OUD presenting to their obstetrician’s office from September 4, 2017, to December 31, 2018, were identified and grouped by mode of OUD treatment delivery: those who received in-person treatment for OUD in their obstetrician’s office (n = 54) and those who received telemedicine for the treatment for OUD in their obstetrician’s office (n = 44) (Figure). *Received treatment* was defined as having attended an initial appointment at the Women’s Reproductive Behavioral Health Program. Race and ethnicity were defined by investigators and reported by participants for the purposes of describing demographic characteristics of the sample.

**Figure.**
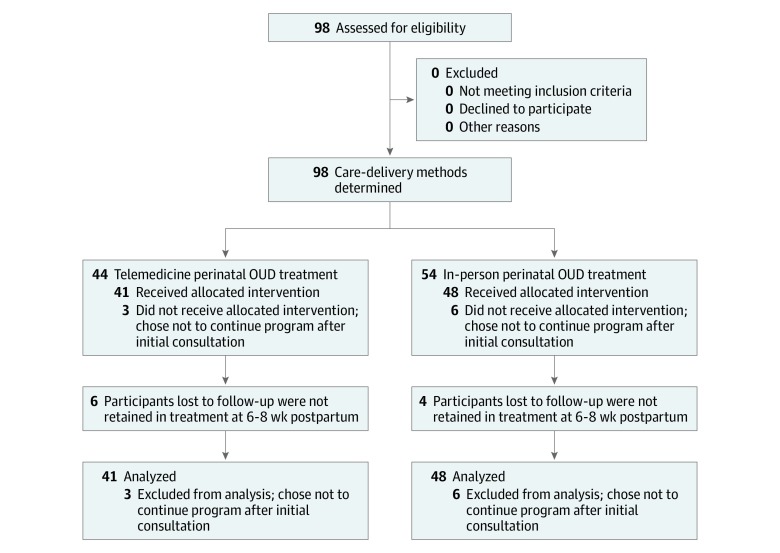
Flow Diagram of Study Participants OUD indicates opioid use disorder.

### Procedures

A standardized clinical protocol for the treatment of perinatal OUD was developed and implemented in both in-person and telemedicine settings (eAppendix 1 in the Supplement). All referred patients were seen in person first per federal and state requirements for prescribing controlled substances (eg, buprenorphine) via telemedicine. Women were seen and evaluated by a psychiatrist with perinatal and addiction training. Pregnant women who received a diagnosis of OUD and were considered appropriate to receive pharmacotherapy for OUD underwent shared decision making^17^ to decide to continue or initiate pharmacotherapy for OUD (ie, buprenorphine). A patient-physician agreement detailing the individual’s treatment plan and frequency of visits as well as understanding of how medication would and would not be prescribed was reviewed and signed by both the patient and clinician (eAppendix 2 in the Supplement). If appropriate, a buprenorphine induction was completed at the time of this first in-person visit. If the patient was appropriate for buprenorphine treatment but not experiencing opioid withdrawal, a buprenorphine induction was completed at home or at a subsequent visit. The obstetricians and addiction care clinicians communicated about each participant via clinical documentation, telephone, and/or telemedicine weekly and as needed.

### Maternal and Newborn Outcomes

The primary maternal outcome was retention in treatment at 6 to 8 weeks post partum, defined as continuous addiction treatment, including uninterrupted treatment with buprenorphine and at least monthly visits with the prescribing psychiatrist during pregnancy through 6 to 8 weeks post partum. Secondary maternal outcomes were positive urine drug screen results, including specific screening for semisynthetic opioids, measured at delivery and 6 to 8 weeks post partum. The primary newborn outcome was NAS documented in the electronic health record. Secondary newborn outcomes included length of newborn hospital stay and birth weight per electronic health record.

### Power Calculation

Power calculations were completed for the primary maternal outcome of retention in treatment and primary newborn outcome of NAS. Assuming a rate of retention in treatment of 0.7500 (75%) under the null hypothesis and 0.8500 (85%) under the alternative hypothesis, group sample sizes of 247 in group 1 and 247 in group 2 were necessary to achieve 80.169% power to detect a difference between the group proportions of 0.1000 (10%). Assuming the rate of NAS to be 0.4000 (40%) under the null hypothesis and 0.5000 (50%) under the alternative hypothesis, group sample sizes of 380 in group 1 and 380 in group 2 were necessary to achieve 80.041% power to detect a difference between the group proportions of 0.1000 (10%).

### Statistical Analysis

Maternal and newborn demographic variables were compared between groups using χ^2^ analyses for dichotomous variables and 2-tailed, unpaired *t* tests or nonparametric Mann-Whitney tests, when appropriate, for continuous variables. In an effort to reduce group selection bias, propensity score methods were used. The propensity score was computed using multiple logistic regression with treatment group (telemedicine vs in person) as the dependent variable. Propensity score and outcome models accounted for demographic and clinical characteristics that potentially could influence treatment group selection or be related to outcome risk. A priori–designated covariates were included in the propensity score model and tested for inclusion in the outcome analyses models to account for a doubly robust analysis method.^18,19^ Covariates included the following baseline characteristics: maternal age, race/ethnicity, socioeconomic status, educational level, tobacco use, benzodiazepine use, number of years of opioid use disorder, and psychiatric diagnoses.

All final analyses were weighted using inverse probability of the treatment weighting approaches to create stabilized weights.^18,20^ The stabilized weight is the marginal probability of being in the telemedicine group given no covariates divided by the propensity score (the probability of being in the in-person group given all covariates). Using inverse probability of the treatment-weighting propensity score methods results in pseudopopulations for each comparison group such that baseline and clinical characteristics listed as potential selection biasing factors are balanced across groups. Balance was assessed using standardized differences and was considered appropriately bias reduced if all covariate differences were less than 0.1 standardized difference.^21^

After the propensity score weights were developed, a series of multivariable models was constructed to make formal statistical comparisons between the telemedicine and in-person groups. For binary outcomes, logistic regression models were used, and for continuous outcomes, linear regression models were used. Poisson and/or negative binomial models were used for count outcomes. Generalized linear models with appropriate link functions (eg, logit, identity, or log) were used for each of these types of models. If any covariates were deemed imbalanced between groups, they were included in the multivariable models. All outcome analysis models include stabilized inverse probability of the treatment weights. Results were reported using bootstrapped estimates and confidence intervals using doubly robust estimation with Proc Causaltrt. SAS/STAT, version 9.4 (SAS Institute Inc) statistical software was used to analyze quantitative data. Findings were considered significant at a 2-tailed *P* ≤ .05.

## Results

A total of 98 pregnant women (mean [SD] age, 30.23 [5.12] years) in 4 outpatient obstetric practices presented for prenatal care at an obstetric practice and were offered addiction services for the treatment of OUD in person (n = 54) or via telemedicine (n = 44). Participants were seen weekly for 4 weeks, every 2 weeks for 4 weeks, and monthly thereafter. Unadjusted baseline demographic characteristics of study participants were similar; however, significantly more women in the telemedicine group reported being married, engaged, or cohabitating with a partner compared with the in-person group (76.1% [35 of 44] vs 53.6% [30 of 54]; *P* = .02) (Table 1). Groups had similar OUD histories, including recent treatment history (Table 1) and similar histories of comorbid substance use and psychiatric diagnoses (Table 2). Although the groups had similar rates of anxiety disorder diagnoses, women seen in person were more likely to be prescribed a benzodiazepine for the treatment of an anxiety disorder at the time of treatment entry compared with those seen via telemedicine (32.14% [18 of 54] vs 10.87% [5 of 44]; *P* = .02) (Table 2).

**Table 1.** Unadjusted Characteristics of Pregnant Women Receiving OUD Treatment via Telemedicine vs in Person^a^

Characteristic	Telemedicine (n = 44)	In Person (n = 54)	*P* Value
Age, mean (SD), y	30.2 (5.5)	30.1 (4.9)	.95
Ethnicity, No. (%)			
Hispanic or Latino	2 (4.3)	4 (7.1)	.69
Race, No. (%)			
White	42 (91.3)	43 (76.8)	.17
Black	2 (4.3)	7 (12.5)	
No. of prior pregnancies, median (IQR)	3.0 (2.0-4.0)	3.0 (1.0-4.0)	.61
No. of living children, median (IQR)	1.0 (0.5-2.0)	1.0 (0.0-2.0)	.44
Fetal gestational age at treatment entry, median (IQR), wk	20.0 (14.0-25.0)	21.5 (13.0-28.0)	.60
Educational level >high school, No. (%)	13 (28.3)	23 (41.07)	.18
Annual household income <$25 000, No. (%)	34 (73.9)	40 (71.4)	.78
Married, engaged, or cohabitating, No. (%)	35 (76.1)	30 (53.6)	.02^b^
Opioid use, No. (%)			
OUD, primarily prescription opioids	31 (67.4)	40 (71.4)	.56
IV prescription opioid use	5 (10.9)	12 (21.4)	.15
OUD, primarily heroin	8 (17.4)	13 (23.2)	.44
IV heroin use	5 (10.9)	12 (21.4)	.15
Cumulative lifetime opioid use, median (IQR), y			
OUD, primarily prescription opioids	5.0 (4.0-7.0)	5.0 (3.0-9.0)	.81
OUD, primarily heroin	3.5 (2.0-5.0)	5.0 (2.0-5.0)	.56
Previous 30-d opioid use			
Daily morphine-equivalent dose of prescription opioids, median (IQR), mg	145.0 (55.0-180.0)	140.0 (60.0-300.0)	.17
OUD pharmacotherapy during previous 30 d, No. (%)			
Buprenorphine	10 (22.7)	15 (27.7)	.26
None	34 (77.2)	39 (72.2)	

Abbreviations: IQR, interquartile range; IV, intravenous; OUD, opioid use disorder.

^a^ Median (IQR) reported for nonnormally distributed data. Percentages are weighted.

^b^ Statistically significant difference.

**Table 2.** Unadjusted Comorbid Psychiatric and Substance Use Disorders Among Pregnant Women Receiving OUD Treatment Via Telemedicine vs in Person

Characteristic	No. (%)		
	Telemedicine (n = 44)	In Person (n = 54)	*P* Value
Current cigarette smoker	10 (21.7)	27 (48.2)	.13
Other substance use disorder			
Alcohol	0	1 (1.79)	>.99
Amphetamine	0	2 (3.57)	.50
Benzodiazepine	0	3 (5.36)	.25
Cocaine	1 (2.17)	4 (7.14)	.37
Marijuana	3 (6.52)	11 (19.64)	.08
Methamphetamine	1 (2.17)	4 (7.14)	.37
Positive UDS at treatment entry, No. (%)	14 (30.4)	22 (39.3)	.35
*DSM-V* diagnosis			
Mood disorder (all types)	27 (58.70)	37 (66.07)	.54
Anxiety disorder (all types)	18 (39.13)	25 (44.64)	.69
Generalized anxiety disorder	9 (19.57)	15 (26.79)	.48
Attention-deficit/hyperactivity disorder	5 (10.87)	5 (8.93)	.75
Chronic pain condition	18 (39.13)	20 (37.04)	.84
Other substance use			
Prescribed benzodiazepine	5 (10.87)	18 (32.14)	.02^a^

Abbreviations: *DSM-V*, *Diagnostic and Statistical Manual of Mental Disorders, Fifth Edition*; OUD, opioid use disorder; UDS, urinary drug screen.

^a^ Statistically significant difference.

### Primary Maternal Outcome: Retention in Treatment

After completing the initial evaluation, 41 of 44 women (93.2%) in the telemedicine group and 48 of 54 women (88.9%) in the in-person group chose to continue treatment in our program. Those who chose to not continue in the program (telemedicine, 3; in person, 6) requested that the consultation be provided to their current addiction clinician for ongoing care. A total of 89 women (telemedicine, 41; in person, 48) opted to continue care in the program. At 6 to 8 weeks post partum, 85.4% (35 of 41) of women in the telemedicine group and 91.7% (44 of 48) of women in the in-person group were retained in treatment (unadjusted *P* = .50) (Table 3). After propensity score weighting and doubly robust estimation controlling for the 2 baseline covariates found to be different between the groups (committed relationship and treatment with benzodiazepines), no significant differences were found between groups (telemedicine: 80.4% vs in person: 92.7%; treatment effect, −12.2%; 95% CI, −32.3% to −4.4%) (Table 3).

**Table 3.** Unadjusted and Adjusted *P* Values for Primary Maternal and Newborn Outcomes Among Pregnant Women Receiving OUD Treatment via Telemedicine vs in Person^a^

Primary Outcome	Unadjusted			Adjusted			
	Telemedicine	In Person	*P* Value	Telemedicine	In Person	Treatment Effect, % (95% CI)^b^	*P* Value
Maternal, No.	41	44	NA	41	44	NA	NA
Newborn, No.	39	45	NA	39	45	NA	NA
Treatment retention 6-8 wk post partum, No. (%)	35 (85.4)	44 (91.7)	.50	(80.4)	(92.7)	−12.2 (−32.3 to −4.4)	.17
NAS, No. (%)	17 (43.6)	28 (62.2)	.12	(45.4)	(63.2)	−17.8 (−41.0 to 8.9)	.12

Abbreviations: OUD, opioid use disorder; NA, not applicable; NAS, neonatal abstinence syndrome.

^a^ Analyses were adjusted for maternal age, race, socioeconomic status, educational level, tobacco use, treatment with benzodiazepines, number of years of OUD, psychiatric diagnoses, and covariates imbalanced between groups; committed relationship; and treatment with benzodiazepines.

^b^ Average bootstrapped treatment effect with 95% bias-corrected CI.

### Primary Newborn Outcome: NAS

We were unable to obtain newborn outcomes for 2 newborns in the telemedicine group and 2 newborns in the in-person group. Within the telemedicine group, 43.6% (17 of 39) of children were born with NAS, while 62.2% (28 of 45) of those in the in-person group had NAS (unadjusted *P* = .12) (Table 3). After propensity score weighting and doubly robust estimation controlling for committed relationship and treatment with benzodiazepines, no significant differences were found between the groups in the proportion of children born with NAS (telemedicine: 45.4% vs in person: 63.2%; treatment effect, −17.8%; 95% CI, −41.0% to 8.9%) (Table 3). A sensitivity analysis that included cigarette smoking as an additional covariate was performed, resulting in the same finding of no significant differences detected between groups.

### Secondary Maternal Outcomes

Positive urine drug screen results at the 6- to 8-week post partum visit were noted in 9.8% (4 of 41) of individuals in the telemedicine group and 20.8% (10 of 48) of individuals in the in-person group (unadjusted *P* = .24) (Table 4). After propensity score weighting and doubly robust estimation controlling for committed relationship and treatment with benzodiazepines, no significant differences were found between the groups. Similar analyses were applied to urine drug screen results at delivery, and no group differences were identified (Table 3). Similar analyses also were applied to the mean number of prenatal and addiction care visits, mean weeks of gestation at delivery, and rates of preterm birth, and no group differences were identified.

**Table 4.** Unadjusted and Adjusted *P* Values for Secondary Maternal and Newborn Outcomes Among Pregnant Women Receiving OUD Treatment via Telemedicine vs In Person^a^

Secondary Outcome	Unadjusted			Adjusted			
	Telemedicine (Maternal n = 41)	In Person (Maternal n = 48; Newborn n = 45)	*P* Value	Telemedicine	In Person	Treatment Effect, % (95% CI)^b^	*P* Value
Maternal, No.	41	44	NA	41	44	NA	NA
Newborn, No.	39	45	NA	39	45	NA	NA
Positive UDS, No. (%)							
At delivery	6 (14.6)	11 (22.9)	.32	(13.2)	(20.6)	−7.5 (−22.7 to 7.8)	.34
At 6-8 wk post partum	4 (9.8)	10 (20.8)	.24	(16.0)	(20.3)	−4.3 (−22.6 to 18.4)	.66
Duration of newborn hospital stay, mean (SD), d	9.1 (7.6)	8.4 (8.3)	.56	9.6	8.6	0.6 (−3.0 to 4.2)	.74
Weight at birth, mean (SD), g	3157.31 (734.03)	2950.19 (635.35)	.24	3117.20	2927.08	190.1 (−106.6 to 486.8)	.21

Abbreviations: NA, not applicable; OUD, opioid use disorder; UDS, urinary drug screen.

^a^ Analyses were adjusted for maternal age, race, socioeconomic status, educational level, tobacco use, treatment with benzodiazepines, number of years of OUD, psychiatric diagnoses, and covariates imbalanced between groups; committed relationship; and treatment with benzodiazepines.

^b^ Average bootstrapped treatment effect with 95% bias-corrected CI.

### Secondary Newborn Outcomes

Unadjusted mean (SD) number of days that the infant was in the hospital following delivery was 9.1 days (7.6) for the telemedicine group and 8.4 (8.3) days for the in-person groups (median, 5 days for both groups, Mann-Whitney *P* = .56). After propensity score weighting and doubly robust estimation controlling for committed relationship and treatment with benzodiazepines, no significant differences were found for days in the hospital following delivery. Similar analyses were applied to birth weight, and no group differences were identified (Table 4).

## Discussion

In this nonrandomized controlled trial, there were no statistically significant differences in rates of retention in treatment and substance use among women receiving virtually integrated OUD treatment within obstetric practices compared with women receiving in-person integrated OUD treatment in an obstetric practice. Similarly, there were no statistically significant differences in the rates of NAS and length of hospital stay among infants whose mothers received integrated OUD treatment via telemedicine compared with in-person integrated OUD care. Although larger and randomized clinical trials with adequate sample sizes are needed, our findings suggest that telemedicine may be one potential solution to integrating addiction treatment into obstetric care, which is especially important for women living in rural areas.

These findings have several public health implications given the detrimental results of untreated perinatal OUD^2,22^ and the benefits of OUD treatment for women and their families.^10^ The most immediate benefit is access to treatment known to reduce the harms of continued drug use in pregnancy^8^ and reduction in maternal mortality.^23^ A state with excellent tracking of maternal deaths recently identified that more than one-third (38.3%) of deaths among women delivering a live infant between 2011 and 2015 were due to opioid-related overdoses,^24^ and in 2014, over 41.4% of maternal deaths were related to substance use.^25^ Nationally, there has been a significant uptick in the number of children placed in foster care owing to parents’ opioid-related deaths, which is largely driven by untreated OUD.^22^ Our study suggests that telemedicine can be used to extend the reach of specialty clinicians necessary to make lifesaving treatments available to pregnant women with OUD and has the potential to reduce maternal mortality and the orphaning of children nationally.

Beyond providing increased access to treatment for OUD, our study suggests that telemedicine can be used to create an integrated model of care. Integrated care that includes obstetric and addiction clinicians who communicate and coordinate a woman’s care in 1 location have demonstrated an increase in treatment retention, improved maternal and newborn outcomes, and cost-effectiveness.^26,27,28^ The rate of retention in telemedicine treatment in our study was similar to that of in-person integrated care (85.4% vs 91.7%) and also similar to that in prior studies of pregnant women receiving treatment for OUD, including methadone or buprenorphine, in integrated treatment centers (70%-80%).^29^ The rates of retention in these programs are higher than those reported in nonintegrated programs, such as methadone treatment centers, where most women, on average, receive only 2.8 consecutive months of methadone treatment before giving birth.^9^ Greater retention and duration of treatment of pregnant women with substance use disorders is associated with a reduction in the use of all substances, including tobacco; an increase in employment; higher income; a reduced likelihood of being arrested; a reduction in symptoms of depression; and more positive parenting attitudes.^30^ In addition, the integration of obstetric and addiction care appears to improve maternal health and functioning^30^ and newborn outcomes,^31,32,33^ including fewer preterm deliveries, infants who are small for gestational age, and infants with low birth weight.^5,6^ Our study suggests that telemedicine can be used to create integrated prenatal and addiction care that has the potential to improve maternal and newborn health.

Our study also noted high rates of poverty and psychiatric comorbidities among pregnant women with OUD, similar to previous studies.^9,34^ While these findings are not novel, they have public health implications. Poverty, maternal mental health, and substance use disorders are leading causes of maternal and infant morbidity and mortality^35^ and can have a significant negative effect on child development.^17,36^ Rural and impoverished communities and counties in the United States with the greatest long-term unemployment and deficit in mental health clinicians are also the counties with the highest rates of NAS.^4^ This finding highlights the complex needs of the mother-infant dyad and underscores the biopsychosocial challenges that this population faces. Telemedicine can be used in obstetric practices to create a comprehensive care setting where women can receive a range of psychiatric, addiction, and psychosocial services in a single location, which is optimal for addressing treatment barriers and needs of this population.^10^

The rates of positive urine drug screen results among women receiving care via telemedicine compared with in-person care were similar. These rates are also similar to those of other studies examining substance use among pregnant women with OUD receiving pharmacotherapy in integrated care settings.^37,38^ In our study, although there was a reduction in the rates of positive urine drug screen results at 6 to 8 weeks post partum compared with treatment entry, continued use of substances occurred despite delivery of evidence-based care. These findings suggest that more effective interventions are necessary for the treatment of perinatal OUD.

The rates of NAS and length of hospital stay in our study were similar to those of prior studies of women receiving buprenorphine for the treatment of OUD.^39^ Neonatal abstinence syndrome is an anticipated and treatable outcome associated with in utero exposure to any opioid, and studies have suggested that buprenorphine compared with methadone for the treatment of perinatal OUD may have a slightly more favorable newborn risk profile,^40,41^ including a lower risk for preterm birth, greater birth weight, and larger head circumference.^41^ In addition, newborns with in utero exposure to buprenorphine compared with methadone require less medication to treat NAS and have a shorter duration of NAS treatment and hospital stay.^39^ If women are eligible for outpatient buprenorphine treatment, increasing access to this treatment via telemedicine may be one way to reduce the short-term costs associated with NAS.

### Limitations

This study has limitations. A randomized clinical trial comparing maternal and newborn outcomes among those assigned to telemedicine vs in-person care would have been an ideal study design to reduce the potential for selection bias. Randomization proved to be challenging as we were unable to provide in-person care in rural clinics owing to costs associated with time, travel, and personnel; thus, propensity score methods were applied. This comparative effectiveness method can result in unbiased effect estimates similar to those of conventional randomized clinical studies^19^; however, unmeasured confounding due to our inability to account for all potential variables remains. In addition, this study was limited by sample size. Although rates of retention in treatment and NAS appear to be similar between the groups, a larger sample is needed to obtain definitive results. This study provides the preliminary data necessary for future, larger-scale randomized clinical trials. A third limitation to this study is that women were followed up only until 2 months post partum. Although women in the study were followed up for an average of 6 months consistent with most addiction treatment studies,^16^ evaluation of pregnant women should extend for a full year post partum, as this period is a critical time for maternal and child health.^25,42^ In addition, the study was conducted in a single state in the southeast United States, and findings may not be generalizable to other states. Replication of findings in other areas of the country may be an important next step in this line of research.

## Conclusions

As the United States faces an epidemic of opioid overdose deaths, access to lifesaving, evidence-based treatment for OUD is necessary. Pregnant women are a particularly vulnerable population in the opioid epidemic owing to significant barriers that impede their ability to access care. Pregnancy, however, is a critical window of opportunity for the treatment of substance use disorders. During this time, women have access to health insurance and are often motivated to reduce their use of substances to invest in the health of their child. Pharmacotherapies, such as buprenorphine and methadone, are effective treatments for OUD and demonstrate greater benefit for pregnant women than no medication or medication-assisted withdrawal.^8^ Making substance use disorder treatment available to pregnant women in which they receive prenatal care may reduce maternal, obstetric, fetal, and newborn morbidity and mortality and will potentially decrease generational transmission of this chronic disease. Telemedicine can provide a scalable solution to making life-saving addiction treatment available to pregnant women in obstetric settings to reduce maternal mortality and improve maternal and child health.

## Notes

Supplement. **eAppendix 1.** Clinical Protocol **eAppendix 2.** Patient-Physician Agreement
