# Supplementary material for: Treatment of Opioid Use Disorder in Pregnant Women via Telemedicine: A Nonrandomized Controlled Trial
Source: JAMA Netw Open. 2020 Jan 31;3(1):e1920177. doi: 10.1001/jamanetworkopen.2019.20177 (PMC7042863; doi:10.1001/jamanetworkopen.2019.20177)
Supplement: Supplement. — eAppendix 1. Clinical Protocol eAppendix 2. Patient-Physician Agreement [file jamanetwopen-3-e1920177-s001.pdf]

Supplementary Online Content

Guille C, Simpson AN, Douglas E, et al. Treatment of opioid use disorder in pregnant women via telemedicine: a nonrandomized controlled trial. *JAMA Netw Open*. 2020;3(1): e1920177. doi:10.1001/jamanetworkopen.2019.20177

eAppendix 1. Clinical Protocol

eAppendix 2. Patient-Physician Agreement

This supplementary material has been provided by the authors to give readers additional information about their work.

© 2020 Guille, C, et al. *JAMA Network Open.*

eAppendix 1: Clinical Protocol

© 2020 Guille, C, et al. *JAMA Network Open.*

Controlled Substance Prescribing via Telehealth for the Treatment of Opioid Use Disorder in Pregnancy

Constance Guille, MD, MSCR
Kathryn King Cristaldi, MD, MHS
James T. McElligott, MD, MSCR

Center for Telehealth, Medical University of South Carolina

© 2020 Guille, C, et al. *JAMA Network Open.*

Controlled Substance Prescribing via Telehealth for the Treatment of Opioid Use Disorder in Pregnancy

Table of Contents

A. Goal 1

B. Background 1

C. Scope of Services 2

D. Protocol for Treatment of Opiod Addiction in Pregnancyvia Telehealth 3

E. Provider Qualifications and Training 5

F. Management of Non-Compliance 5

G. Interdisciplinary Team and Roles 6

H. Inegration with Obstetrical Home 6

I. Duration of Management and Transfer of Care 7

J. Quality Assurance and Reporting 7

K. Patient Safety and Emergency Plan 7

L. Providers Participating 8

M. Current Participating Obstetric Practices and Locations 9

Appendix I 10

© 2020 Guille, C, et al. *JAMA Network Open.*

Controlled Substance Prescribing via Telehealth for the Treatment of Opioid Use Disorder in Pregnancy

A. Goal

To decrease the occurrence of illicit opioid use during pregnancy and decrease the severity of neonatal abstinence syndrome through the expansion of access to addiction medicine specialists trained in the best practices for diagnosis, tapering of medications and treatment of opioid use, misuse or addiction during pregnancy.

B. Background

The use of illicit opioid medication in pregnancy has been steadily on the rise over the past five years with one study estimating the number of pregnant women with substance use disorders associated with these drugs climbing five-fold from 2000-2009. According to the 2010 National Survey on Drug Use and Health, an estimated 4.4% of pregnant women reported illicit drug use in the past 30 days. An accompanying proportionate increase in the rate of infants born with neonatal abstinence syndrome has also been observed nationally and in South Carolina rates of NAS reached 3.9 infants per 1,000 births in 2012. The American College of Obstetricians and Gynecologists supports the use of opioid-assisted therapy during pregnancy to prevent complications of illicit opioid use and narcotic withdrawal, encourage prenatal care and drug treatment, reduce criminal activity, and avoid risks to the patient of associating with a drug culture. Comprehensive opioid-assisted therapy that includes prenatal care reduces the risk of obstetric complications. While neonatal abstinence syndrome is an expected outcome of this treatment the use of buprenorphine has been associated with neonates requiring lower doses of medication, shorter duration of treatment and fewer days of hospitalization.

Currently there are addiction treatment centers in South Carolina, however, lack of transportation, lack of education, stigma and economic barriers to this care exist and contribute to racial and socioeconomic disparities in addiction and rates of neonatal abstinence syndrome. In accordance to the South Carolina Telemedicine Act, practicing via telemedicine is held to the same standard of care as licensees employing more traditional in-person medial care. The prescribing of controlled substances is prohibited in the setting of telemedicine unless an exception is granted by the SC Board of Medical Examiners.

Addiction and pain medicine specialists that work specifically with pregnant and postpartum women are available to connect via telehealth to patients located in their primary obstetrician's office and these visits include an on-sight obstetrician. This telehealth program that assists with curbing opioid misuse behaviors and tapering opioid medication during pregnancy is currently active with prescribing deferred to the obstetrical provider. However, this fragmentation of care plan is suboptimal and places a burden on the primary obstetricians as they are commonly uncomfortable providing the recommended treatment. Further the majority of obstetricians do not have a buprenorphine license and are unable to prescribe this treatment for pregnant women with opioid use disorder (OUD). Pregnant women with OUD who are candidates to start buprenorphine are required to travel to Charleston to be seen in person before starting buprenorphine treatment. Even if obstetricians become licensed to

© 2020 Guille, C, et al. *JAMA Network Open.*

prescribe buprenorphine, there are still barriers to delivering the standard of care for this population. Specifically, the primary obstetric providers:

- Are not able to provide addiction counseling which needs to be delivered with buprenorphine treatment;
- Are not able to provide other non-opioid pain management strategies;
- Do not feel comfortable/are not familiar with risk management strategies to monitor prescription opioid or buprenorphine use, misuse or abuse.

C. Scope of Services

The potential diagnosis/condition treated is opioid use, misuse, opioid addiction in pregnancy as well as reduction of the severity of neonatal abstinence syndrome. The proposed protocol involves the treatment of these opioid use, misuse and opioid use disorder using telehealth technology to connect an addiction medicine specialist to pregnant patients while the patient remains at their local obstetric home as much as possible. The standard of care for the management of opioid use, misuse and use disorder will be maintained. Of note, incorporation of telehealth allows for the use of best practices in the management of opioid use, misuse and opioid use disorder which are otherwise difficult to attain for the majority of practice settings in the state. These best practices incorporate counseling related to the use of medications in pregnancy, motivational enhancement to decrease use, misuse and abuse, therapy, medication tapering and monitoring risk associated with meds (misuse, abuse, diversion etc.). Importantly, relapse prevention therapy is critical aspect of an optimized care plan.

The intent of this initiative is to increase access to specialized providers via telehealth to be used as an adjunct to, but not substitute for, in-person care in the treatment of opioid use, misuse and substance use disorder.

Goals of Treatment of Opioid Addiction in Pregnancy via Telehealth:

- Increase access to addiction specialty care for pregnant women, enabling women across the state to receive treatment in accordance with the standard of care to include addiction counseling, therapy, medication monitoring and relapse prevention.
- Decrease barriers to addiction treatment such as stigma, misinformation, transportation costs, loss of work and productivity and time away from family and support services.
- Ameliorate racial and socioeconomic and rurality based disparities in addiction services for pregnant South Carolinians as well as similar disparities in infants born with neonatal abstinence syndrome.
- Alleviate the burden of treating opioid addicted pregnant women on local family practice, obstetric and emergency providers.

© 2020 Guille, C, et al. *JAMA Network Open.*

D. Protocol for Treatment of Opioid Addiction in Pregnancy via Telehealth

1. Patient Referral and Initial Assessment

Patients referred for treatment of opioid addiction in pregnancy will be referred to the program by PCP, OB, emergency services or addiction treatment program. Self-referrals are also permissible. Initial assessment will be preformed in-person to establish appropriate diagnosis and care. At this time a Patient-Physician Agreement detailing the individual's treatment plan and frequency of visits as well as understanding of how medications will and will not be prescribed (see Appendix 1 for Patient Physician Agreements) for the prescribing of buprenorphine or opioid medications will be signed by both parties.

2. Parameters for Follow-up Care

In-person follow up visits will be preformed every six months for the duration of treatment. Additional visits will be conducted using telehealth technology to include secure, HIPPA compliant video conferencing to connect the provider to the patient while they are located in their local obstetrician's office. These visits will be scheduled, whenever possible, in conjunction with an obstetrical visit. Follow-up visits via telehealth technology will include medication management and therapy to address addiction, relapse prevention as well as pain and/or other comorbid mental health problems as appropriate. Follow-up care frequency initially is weekly and may be gradually stepped down as appropriate but would never be less frequent than once per month. At each follow-up visit opioid use, misuse and abuse is evaluated using the prescription drug monitoring program (every visit), urine drug screens (at least once per month), objective and patient-report subjective assessments of opioid use, cravings, misuse and abuse (every visit).

3. Method of verification of patient and location

Patient identification will be verified at the time of registration and check-in at the local originating office. Once the patient has been placed in an examination room and telehealth connection with specialty provider has been established, a trained tele presenter will again verify the patient identification and location verbally to the telehealth provider. Future capabilities include verification of patient location through telehealth scheduling and connecting platform.

4. Telehealth visit

The telehealth visit should include but is not limited to the following:

© 2020 Guille, C, et al. *JAMA Network Open.*

- Assessment, to include:
    - Review of the Prescription Drug Monitoring Program
    - Assessment of patient’s pain, affect, activity and adjunctive treatments
    - Screens for aberrant behaviors (e.g., substance misuse) and substance use disorders
    - Review of pertinent medical and psychiatric history
    - Review of medications and pertinent labs
    - Mental status examination
- Consultation and Plan of Care to include:
    - A Patient-Physician Agreement (See Appendix 1)
    - Discussion of risks and benefits of treatment including medication(s)
- Counseling, to include:
    - Motivational Enhancement Therapy
    - Relapse prevention therapy
    - Cognitive Behavioral Therapy

5. <u>Prescribing of controlled substances to treat addiction</u>:

Medications to be prescribed include buprenorphine (Suboxone) and opioid medications limited to the setting of chronic disease management during pregnancy. Prescribing will be in accordance with the South Carolina Telemedicine Act and the Controlled Substances Act and will participate in and utilize the Prescription Drug Monitoring Program at each instance of prescribing the controlled substance. Prescriptions for prescription opioids will be mailed directly to the pharmacy of choice and buprenorphine will be phoned in to the pharmacy of choice. Confirmation of failed delivery of prescription opioids will be obtained. Future capabilities will enable electronic prescriptions compliant with applicable regulations.

6. <u>Elements of the Patient-Physician Agreement</u>

a. All controlled substances must come from your provider or, during his/her absence, by the covering physician, unless specific authorization is obtained for an exception.
b. Routine assessment of your functioning will be conducted.
c. Urine or serum toxicology screens will be conducted.
d. A pill-count may be requested to ensure you are taking prescribed doses.
e. Medications may not be replaced if they are lost, stolen, or destroyed.
f. Early refills will not be given.
g. You may not share, sell, or otherwise permit others to have access to these medications.
h. We recommend using a lockbox to safeguard these medications.

© 2020 Guille, C, et al. *JAMA Network Open.*

i. Urine drug screen at initiation and monthly during ongoing treatment

7. Provider Documentation

Licensees prescribing controlled substances must maintain a complete record of the telemedicine patient's care according to prevailing medical record standards in accordance with the Board's Telemedicine Advisory Opinion. In addition, the provider should document utilization of the Prescription Monitoring Program and a completed patient/physician controlled substances agreement within the medical record.

8. Exclusion Criteria

a. Upon initial in-person consultation, screening and diagnosis patients who meet the following criteria will not be eligible for further consultation via telehealth:
b. Unwilling or unable to attend in-person visits every 6 months
c. Unwilling to agree to any element of the Patient Physician Agreement
d. Evidence of any past or current opioid diversion (e.g., selling prescribed opioid medications or controlled substances)
e. Unwilling or unable to attend recommended telehealth appointments
f. Unwilling or unable to attend recommended prenatal care appointments

E. Provider Qualifications and Training

All those providers prescribing controlled substances via telehealth for the treatment of addiction/pain control in pregnancy will:

- receive privileging at MUSC to include training on SC Telemedicine Act, Controlled Substances Act and equipment use
- practice according to standard DEA and state requirements
- participation in the prescription drug monitoring program

F. Management of Patient Non-Compliance

Patients who are not able to be compliant with treatment regimen or are found to have breached the Physician-Patient Agreement will be returned to in-person models of care. An appropriate referral to an in-person treatment center will be made. The telehealth team will continue to facilitate this referral until in-person treatment is fully established. All providers will be kept up-to-date with patient's treatment referral and engagement.

© 2020 Guille, C, et al. *JAMA Network Open.*

G. Interdisciplinary team and roles:

Connecting provider

- Connect to the patient at designated time and conduct visit per aboveprotocol
- Communicate all treatment plans to primaryobstetrician
- Be available to primary obstetrician via telehealth video conferencing, email or phone 24/7 in the event of patient emergency.

Role of the Originating Site

1. On site physician:
- provide regular obstetrical services
- communicate with connecting physician regarding treatment plans, pertinent test results, aberrant patient behaviors

2. Administrative staff
- Identify patient using state-issued identification and register according tothe workflow of the originating site

3. Telepresenter
- Complete telepresenter training
- Identify patient and verify patient identity and location to connecting provider
- Use telemedicine equipment with connecting provider as trained
- Assist with scheduling and obtaining results forpatients
- Assist with office vitals and medication reconciliation

H. Integration with Obstetrical Home

1. Communication:

The primary obstetrician will supply all necessary patient records to the addiction medicine specialist at the time of referral. Following the initial evaluation with the addiction specialist, a consultation letter detailing the patient's diagnosis and plan or care by the addiction medicine physician will be shared with the primary obstetrician with periodic follow-up letters regarding any deviation from the original plan. Any orders suggested by the specialist will be clearly communicated in writing for the review of the primary obstetrician via consult note.

2. Emergency Access Plan:

In the event of patient emergency identified by the addiction medicine specialist will be communicated immediately via phone or videoconferencing to the obstetric provider. In the event of a patient emergency identified by the obstetric provider related to addiction, the addiction specialist will be available for consultation with the obstetrical home via phone or video conferencing at all times (24 hrs./day, 7days/week).

© 2020 Guille, C, et al. *JAMA Network Open.*

3. Collaborative Process Improvement

Quality metrics such as patient retention and control of symptoms, success in weaning of opioid medications as well as surveys of provider and patient satisfaction will be collected. These quality metrics, combined with a comparison of treatment data with historical data will be evaluated and shared with the treatment team who will then engage in collaborative process improvement.

I. Duration of management and transfer of care

Treatment will continue for the duration of pregnancy with a plan for transfer of care to a local addiction medicine specialist, or pre-pregnancy pain management provider during the post partum period. Local providers and other support services will be identified at the time of treatment initiation with plans for transition of care made and clearly communicated to all parties at treatment initiation and throughout the course of pregnancy. At the time of transfer of care all records and care plans will be provided to the local provider and other local support services will be notified. Future capabilities could include continuation of addiction services into the post partum period pending program development approval by the Board of Medical Examiners. When accepting a referral for the prescribing of opioid medication during pregnancy for a patient whom a physician who has been prescribing opioid medication prior to pregnancy, a plan for transfer of care back to the original prescribing physician within three months following delivery will be established and a patient-physician agreement (Appendix I) as well as an executed contract with the primary care provider (Appendix II) reflecting this agreement will be signed by all parties.

J. Quality Assurance and Reporting

The MUSC program will report quality data (see section H.3 above) and comparison of treatment data with historical treatment data for this patient population to the Board of Medical Examiners every 6 months.

K. Patient Safety and Emergency Plan

1. Obstetrician/Connecting providercommunication

- The connecting provider will inform the referring Obstetrician via phone or videoconferencing of reason and plan for transfer to ED. Office staff will assist with the transfer of patient to ED per their in-person protocol.

© 2020 Guille, C, et al. *JAMA Network Open.*

2. Emergency Transfer Coordination

- The ED will be provided with the following in writing:
  - Name and contact information for the primary obstetrician and connecting addiction medicine specialist
  - Full medication reconciliation and treatment plan
  - Any additional information or processes as requested by the ED leadership
  - A signed letter of acknowledgement/agreement from the local ED to accept patients will be obtained

3. In-Clinic Emergency Procedures:

- In the event of a medical emergency during telehealth consultation the tele-presenter will communicate the emergency to clinic staff and the clinic's emergency protocol shall apply
- The connecting addiction medicine specialist will provide all medication and treatment information to those responding to the emergency and will be available via teleconferencing for consultation.
- In the event of an in-clinic emergency prior to the initiation of a telehealth visit, the clinic staff shall initiate the clinic emergency protocol and the connecting addiction medicine specialist should be informed and available for consultation.

4. Examples of Clinical Parameters Indicating Need to Initiate Immediate Referral to Emergency

- In the event of opioid intoxication or severe opioid withdrawal symptoms, a transfer to a local Emergency Department will be facilitated.
- In the event of imminent risk to self or others, a transfer to a local Emergency Department will be facilitated.

L. <u>Providers Participating</u>

The MUSC program requesting this exemption is led by Dr. Connie Guille an Associate Professor in the Department of Psychiatry and Behavioral Sciences at MUSC and director of the Women's Reproductive Behavioral Health Program at MUSC. Dr. Guille completed her Psychiatry Residency training and subspecialty training in Perinatal Psychiatry at Yale University. She is the recipient of two National Institute of Health career development awards aimed at reducing perinatal opioid use, misuse and abuse. She has established strong collaborations with the department of Obstetrics and Gynecology at MUSC and with obstetricians locally as well as throughout the state to address opioid use in pregnancy.

Providers and Buprenorphine registration numbers include:

- Constance Guille, MD (XG 1593195)
- Kelly Barth, DO (XS 7993341)
- Lisa Boyars, MD (XB 4007539)

M. Current Participating Obstetric Practices and Locations

McLeod Hospital, Florence, SC
Carolina Ob/Gyn, Murrells Inlet, SC
Magnolia Ob/Gyn, Myrtle Beach, SC

© 2020 Guille, C, et al. *JAMA Network Open.*

eAppendix 2:
Patient-Physician Agreement

© 2020 Guille, C, et al. *JAMA Network Open.*

The purpose of this agreement is to provide you with the best possible treatment for your Opioid Use Disorder and to protect your access to buprenorphine or buprenorphine/naloxone medications and addiction services. Your treatment plan of care and response to treatment will be communicated with your referring obstetrics provider.
There are clear benefits with the combined treatment of buprenorphine or buprenorphine/naloxone medication and psychotherapy to treat opioid addiction. If it is determined by your physician that you are a candidate for buprenorphine or buprenorphine/naloxone medication you will be required to attend regular medication management appointments and psychotherapy for relapse prevention appointments with a frequency determined by you and your provider as detailed below. The frequency of these appointments will be as follows, unless your provider gives you an alternative frequency. Following the start of buprenorphine or buprenorphine/naloxone treatment you will be required to attend appointments as designated by your provider weekly for medication management and weekly psychotherapy for 4 weeks. After 4 weeks, if appropriate, you will then be required to attend these appointments every two weeks. After 4 weeks, if appropriate, you will then be required to attend these appointments monthly. You will be required to attend an in-person appointment at the initiation of treatment and every six months.

_________/________
Patient/Provider Initials

Failure to attend your medication management appointments or psychotherapy appointments or required in-person visits, can result in termination of treatment from this clinic. If you need to cancel an appointment, this must be done in advance of 24 hours prior to your scheduled appointment. Please call [provider phone number]: _ if you need to cancel your appointment. Failure to attend or not cancel an appointment in advance on 3 occasions will result in termination of your care at this clinic and referral to another treatment center for your Opioid Use Disorder.

_________/________
Patient/Provider Initials

While there are clear benefits with the use of buprenorphine or buprenorphine/naloxone, there are also clear risks with the use of these medications including unintentional overdose and death when combined with other legal or illegal drugs such as alcohol, opioids (narcotic analgesics), benzodiazepine tranquilizers, barbiturate sedatives and other illegal drugs. In

© 2020 Guille, C, et al. *JAMA Network Open.*

addition, because these drugs have potential for abuse or diversion, strict accountability is necessary when use is prolonged. For this reason the following policies are agreed to by you, the patient, as consideration for, and a condition of, the willingness of your physician to consider the initial and/or continued prescription of buprenorphine or buprenorphine/naloxone to treat your opioid addiction.

1. All buprenorphine or buprenorphine/naloxone prescription must come from your provider or, during his/her absence, by the covering physician, unless specific authorization is obtained for an exception. Multiple sources can lead to untoward drug interactions or poor coordination of treatment.
2. The use of other controlled substances such as opioids (narcotic analgesics), benzodiazepine tranquilizers, barbiturate sedatives are prohibited, unless specific authorization is obtained for an exception.
3. The use of illicit substances such as marijuana, cocaine, heroin, or amphetamines is prohibited.
4. Your provider and his/her team have permission to discuss all diagnostic and treatment details with dispensing pharmacists or other professionals who provide your health care for purposes of maintaining accountability and coordinatingcare.
5. You may not share, sell, or otherwise permit others to have access to these medications. Should an egregious violation of state or federal law occur there will be discontinuation of these services.
6. Unannounced urine or serum toxicology screens may be requested, and your cooperation is required. Presence of unauthorized substances may prompt referral for a higher level of addictions treatment (e.g., intensive outpatient or inpatient treatment) and, may result in cessation of buprenorphine or buprenorphine/naloxone medications by your physician, or may result in discontinuation of services completely.
7. Prescriptions and bottles of these medications may be sought by other individuals with chemical dependency and should be closely safeguarded. It is expected that you will take the highest possible degree of care with your medication and prescription. They should not be left where others might see or otherwise have access to them. We suggest having a lockbox for your medication(s).
8. At any time between visits to the clinic, any member of the treatment team may require you to come to a local clinic and bring all of your medications for a pill-count to ensure you are taking prescribed doses. Failure to come in when requested, or presenting to the clinic with fewer pills than you should have left at the time of the pill-count may result in cessation of buprenorphine or buprenorphine/naloxone medications by your physician, referral for further specialty assessment, or discontinuation of servicescompletely.
9. Since these drugs may be hazardous or lethal to a person who is not tolerant to their effects, especially a child, you must keep them out of reach of such people. We suggest having a lockbox for your medication(s).
10. Medications may not be replaced if they are lost, stolen, get wet, are destroyed, left on an airplane, etc. If your medication has been stolen you can complete a police report regarding the theft.
11. Early refills will not be given.

© 2020 Guille, C, et al. *JAMA Network Open.*

12. Prescriptions may be issued early if the physician or patient will be out of town when a refill is due. These prescriptions will contain instructions to the pharmacist that they not be filled prior to the appropriate date.
13. Renewals of buprenorphine or buprenorphine/naloxone and continued participation in this program are contingent on keeping all scheduled medication and therapy appointments.
14. You may not phone for prescriptions after hours or on weekends.
15. It is understood that any medical treatment is initially a trial, and that continued prescription is contingent on evidence of benefit. Routine assessment of your functioning will be conducted and you are required to participate in theseassessments.
16. It is understood that failure to adhere to any of these policies may result in cessation of buprenorphine or buprenorphine/naloxone therapy by your physician, referral for further specialty assessment, ordiscontinuation of our services.
17. You affirm that you have full right and power to sign and be bound by this agreement, and that you have read, understand, and accept all of itsterms.

Patient/Participant Signature	Type Patient/Participant Name	Date
Clinician Signature	Type Clinician Name	Date

© 2020 Guille, C, et al. *JAMA Network Open.*
